# Choroidal neovascular membrane in intraocular tuberculosis

**DOI:** 10.3205/oc000075

**Published:** 2017-09-01

**Authors:** Koushik Tripathy

**Affiliations:** 1 Department of Vitreoretina and Uvea, ICARE Eye Hospital & Postgraduate Institute, Noida, Uttar Pradesh, India

**Keywords:** intravitreal anti-VEGF agents, tuberculoma, tubercular subretinal abscess, Avastin, bevacizumab

## Abstract

Recently, Lodhi SA and colleagues have reported on a patient with choroidal neovascular membrane (CNVM) associated with tubercular granuloma, who responded to intravitreal bevacizumab injections. The available literature regarding CNVM associated with intraocular tuberculosis is briefly discussed. Choroidal neovascular membrane is a possible complication of choroidal tuberculosis and the patients should be informed about this risk. However, patients usually respond well to intravitreal anti-vascular endothelial growth factor agents as evidenced by the case reported by us and the current case reported by Lodhi et al.

## Letter to the Editor

Dear Editor,

I read with interest the report on choroidal neovascular membrane (CNVM) [1] in intraocular tuberculosis (IOTB) which responded to 4 bevacizumab injections. I humbly want to discuss a few facts.

**Figure 1:**
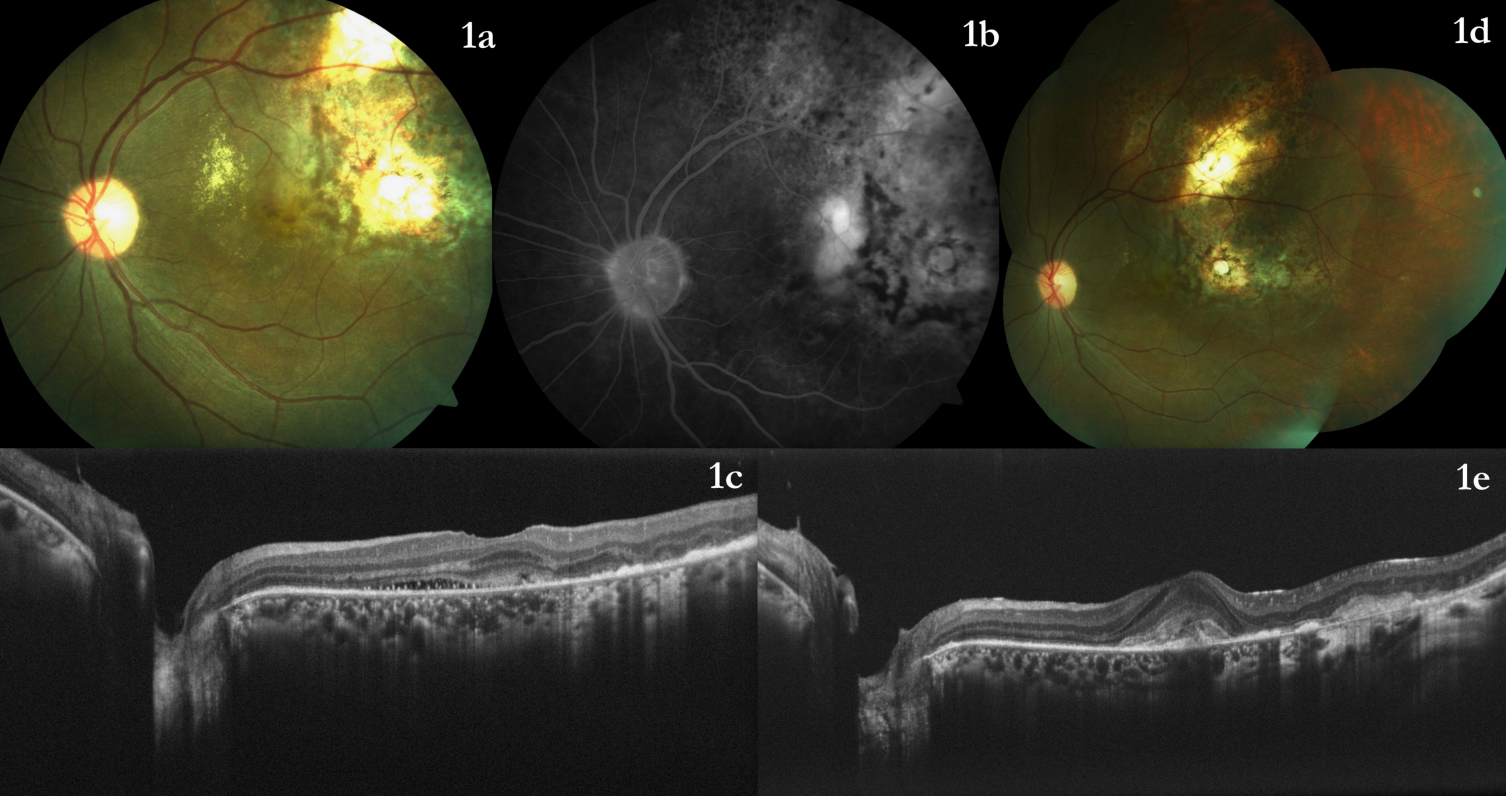
a) The left eye showed a healed choroidal tuberculoma with macular hard exudates, subretinal fluid and subretinal bleed. b) The fluorescein angiogram showed an active choroidal neovascular membrane. c) The optical coherence tomography (OCT) showed subretinal fluid. d) At 1 month, the hard exudates had reduced considerably and subretinal bleed had resolved. e) The optical coherence tomography showed a subretinal scar with no intraretinal or subretinal fluid at any macular scan. (Modified from [4] with kind permission from Taylor and Francis.)

1. The article emphasizes the paradigm that any disease which disturbs the retinal pigment epithelium and Bruch’s membrane can lead to a CNVM. The increased vascular endothelial growth factor (VEGF) levels and hypoxia within the choroidal tubercular granuloma have been reported which may play a role in CNVM formation in such cases [2]. However, CNVM in choroidal tuberculoma is not an unknown entity.
  We had reported the case of a 36-year-old female [3] suffering from pulmonary tuberculosis (TB) who had a large choroidal tuberculoma. The patient was treated with anti-tubercular treatment (ATT) for active pulmonary tuberculosis. She also received oral steroid under the cover of ATT, following which pulmonary tuberculosis was cured and the choroidal tuberculoma healed resulting in chorioretinal scar. She achieved a best-corrected visual acuity (BCVA) of 6/12 after the tubercular granuloma healed. However, her vision declined 1 year after the completion of ATT due to a CNVM [4] with subretinal bleed, hard exudates, and subretinal fluid at fovea at the edge of the chorioretinal scar (Figure 1a (Fig. 1)). At this time, there was no active choroidal or intraocular inflammation. On systemic investigations and physician review, no evidence of active pulmonary tuberculosis was noted. Optical coherence tomography showed subretinal fluid and CNVM (Figure 1c (Fig. 1)), and the fluorescein angiogram revealed an active membrane (Figure 1b (Fig. 1)) [4]. She responded well to one intravitreal injection of bevacizumab with resolution of subretinal fluid (Figure 1 d,e (Fig. 1)) and regained a BCVA of 6/18, which she maintains till date. At the time of detection of CNVM, there was no evidence of active TB (systemic or intraocular) or active intraocular inflammation. So, we did not repeat the anti-tubercular treatment and also we did not start systemic steroid. The intraocular neovascularization process (CNVM) was treated successfully with anti-VEGF agent alone.
  In indexed peer-reviewed literature, probably the earliest case of CNVM in IOTB had been reported by Chung YM and colleagues [5] in 1989. This patient (54-year-old Chinese lady) had tubercular meningitis, multiple choroidal tubercles, and disciform maculopathy [5]. Gupta V and coauthors had reported another patient (23-year-old male) with epididymo-orchitis with choroidal tuberculoma who later developed a CNMV [6].
  Thus, all patients with choroidal tuberculosis should be explained about the risk of CNVM and should be kept on follow-up. However, such patients usually respond favorably [1], [4] to anti-VEGF agents.
2. The histopathological report of the hysterectomy specimen may be relevant in this case, and it would be interesting to know the result of the same if it was available.
3. Choroidal tuberculosis is usually bilateral in disseminated tuberculosis [7]. Though a unilateral presentation is possible, minute indirect ophthalmoscopic examination of the fellow eye is important to rule out small tubercles which may even be present in the mid-periphery.

## Notes

### Competing interests

The author declares that he has no competing interests.
